# Safe and successful birth following pelvic radiotherapy for rectal mucosa-associated lymphoid tissue lymphoma: a case report

**DOI:** 10.1186/s13256-016-1193-z

**Published:** 2017-02-01

**Authors:** Yoshiomi Hatayama, Masahiko Aoki, Hideo Kawaguchi, Katsumi Hirose, Mariko Sato, Hiroyoshi Akimoto, Mitsuki Tanaka, Ichitaro Fujioka, Shuichi Ono, Yoshihiro Takai

**Affiliations:** 1 Department of Radiology and Radiation Oncology, Hirosaki University Graduate School of Medicine, 5 Zaifu-cho, Hirosaki, Aomori 036-8562 Japan; 2 Southern Tohoku BNCT Research Center, Koriyama, Japan

**Keywords:** Rectal MALT lymphoma, Radiotherapy, Ovarian function, Natural pregnancy, Safe birth

## Abstract

**Background:**

Mucosa-associated lymphoid tissue lymphomas can occur in various parts of the body, and half of mucosa-associated lymphoid tissue lymphomas occur in the gastrointestinal tract. Gastric mucosa-associated lymphoid tissue lymphoma is the most common lymphoma of the gastrointestinal tract and primary rectal mucosa-associated lymphoid tissue lymphoma is very rare. Because of the high radiosensitivity of mucosa-associated lymphoid tissue lymphomas, this condition can be controlled with radiotherapy of approximately 30 Gy alone. However, ovarian dysfunction as an adverse event of radiotherapy for pelvic lesions can become a problem in girls and women. We report a case of a 28-year-old woman with rectal mucosa-associated lymphoid tissue lymphoma who safely gave birth to a baby following 30.6 Gy radiotherapy to her whole rectum.

**Case presentation:**

A 28-year-old Japanese woman became aware of bloody stools and was diagnosed as having Lugano I rectal mucosa-associated lymphoid tissue lymphoma. She was referred to our institute and initiated on radiotherapy. However, she expressed a desire to bear children. We used horizontally opposed pair fields for radiotherapy to minimize the irradiation to her endometrium and ovary. A total dose of 30.6 Gy was given in 17 fractions of 1.8 Gy by 10-Megavolt X-ray linear accelerator. As a result, one-third of her uterus and half of her ovary were outside the irradiation field. After approximately 1 year of treatment, positive pregnancy was confirmed and finally she safely gave birth to a baby girl without congenital abnormalities.

**Conclusions:**

This report provides hope for girls and women who have undergone irradiation for pelvic mucosa-associated lymphoid tissue lymphomas and who desire to bear children.

## Background

Mucosa-associated lymphoid tissue (MALT) lymphoma is a low-grade B cell lymphoma, first identified by Isaacson and Wright in 1983 [1]. This lymphoma can occur in various parts of the body, and half of all MALT lymphomas occur in the gastrointestinal tract. Gastric MALT lymphoma is the most common gastrointestinal tract lymphoma, comprising 85% of all cases, and primary rectal MALT lymphoma is very rare [2, 3]. Because of the low frequency of the disease, the optimal therapeutic strategy for primary non-gastric lymphomas is yet to be established; however, prognosis is good, and the 5-year and 10-year overall survival rates have been reported to be approximately 90% and 80%, respectively [4, 5]. Radiotherapy or surgical resection is considered to be a therapeutic strategy of limited stage MALT lymphomas, but because of the high radiosensitivity of MALT lymphomas, this condition can be controlled by radiotherapy of approximately 30 Gy alone [6–8]. The present case was of a 28-year-old Japanese woman who desired to bear children. However, ovarian dysfunction as an adverse event of radiotherapy for pelvic lesions can become a serious problem. Here we report a case of a 28-year-old woman with rectal MALT lymphoma who safely gave birth to a baby following 30.6 Gy radiotherapy to her whole rectum.

## Case presentation

**Fig. 1:**
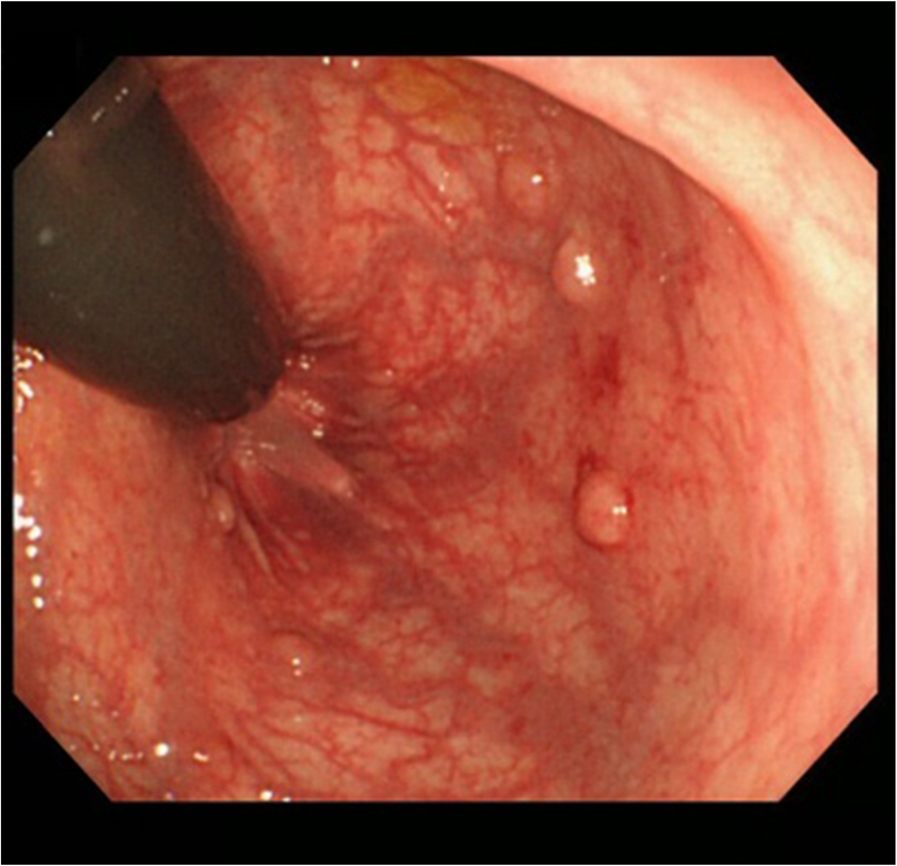
Colonoscopy showing elevated lesions with mild bleeding in the Rb section of the rectum

A 28-year-old Japanese woman became aware of bloody stools from August 2013 and visited a neighborhood clinic dedicated to internal medicine and digestive organs in September 2013. An initial examination by colonoscopy identified elevated lesions with mild bleeding in the Rb section of her rectum (Fig. 1). These rectal lesions were biopsied for analysis. Immunohistochemical investigation of the biopsy revealed that the proliferating cells were phenotypically characterized as CD20+, CD79a+, CD3−, CD5−, CD10−, and cyclin D1−. Based on these findings, she was diagnosed as having MALT lymphoma. A complete blood count revealed a hemoglobin level of 10.6 g/dl. Biochemical examination of her blood showed a lactate dehydrogenase (LDH) level of 102 IU/l and a soluble interleukin-2 receptor (sIL-2R) level of 190 U/ml (reference value, 145 to 519 U/ml). Although her hemoglobin was slightly reduced, LDH and sIL-2R were within the normal range. She was referred to the Department of Gastroenterology of a general hospital in October 2013. No enlargement of regional nodes or distant metastasis was revealed by positron emission tomography-computed tomography (PET-CT) and contrast-enhanced computed tomography (CT). Finally, she was diagnosed as having Lugano I rectal MALT lymphoma.

She was then introduced to our institute and initiated on radiotherapy. However, she expressed a desire to bear children and had some embryos frozen by the Department of Obstetrics and Gynecology before being admitted to our department in February 2014.

**Fig. 2:**
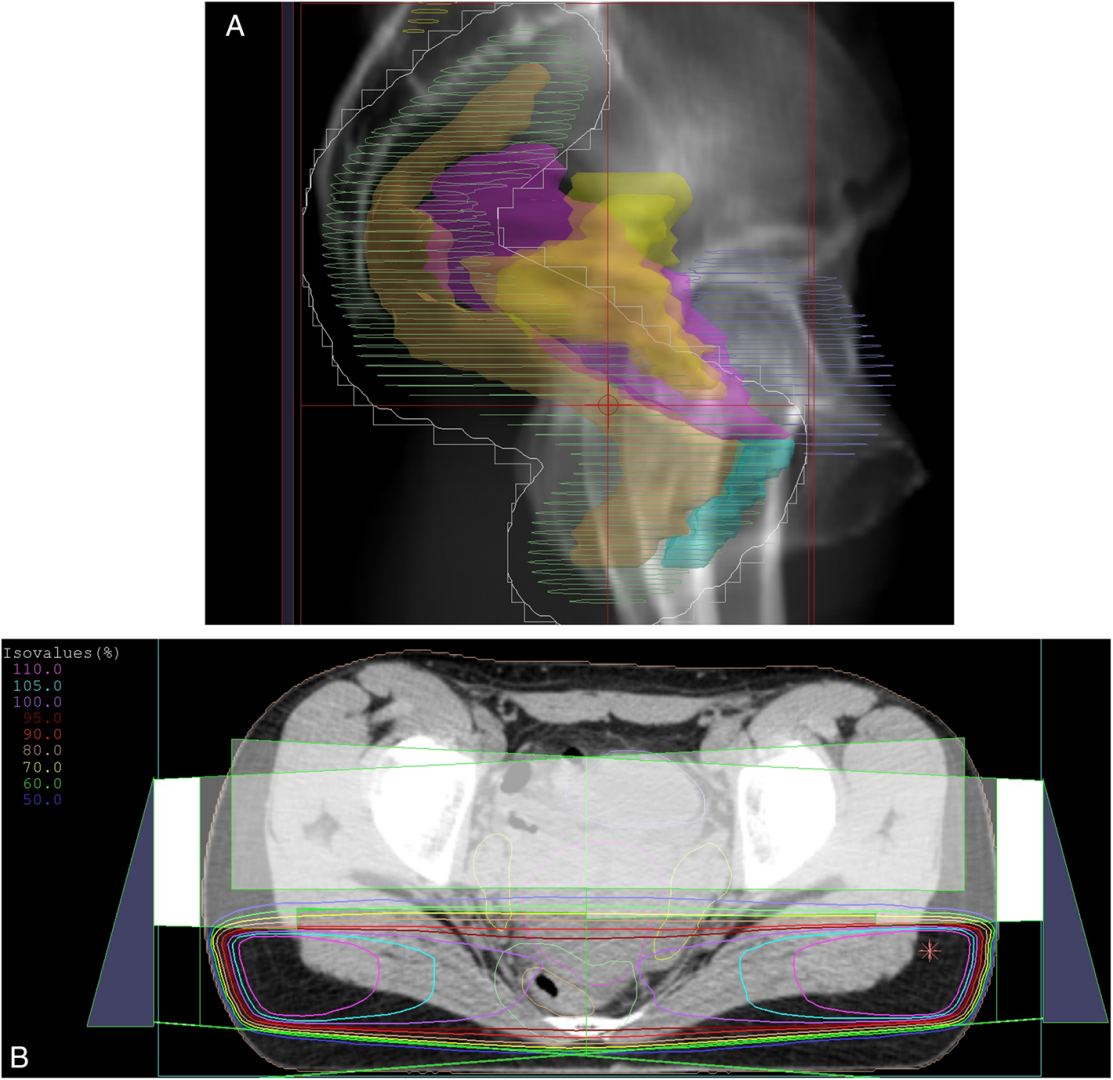
**a** The irradiation field. Clinical target volume was defined as the whole rectum and is shown in *orange*. Planning target volume was defined as clinical target volume plus a 1 cm margin in all directions and is shown in *green*. The ovary and uterus are shown in *yellow* and *pink*, respectively. **b** Dose distribution of the axial cross section. Although a hot area of more than 110% spread to both sides of the soft tissue, the ventral side of the ovary and uterus remained outside the irradiation field

**Fig. 3:**
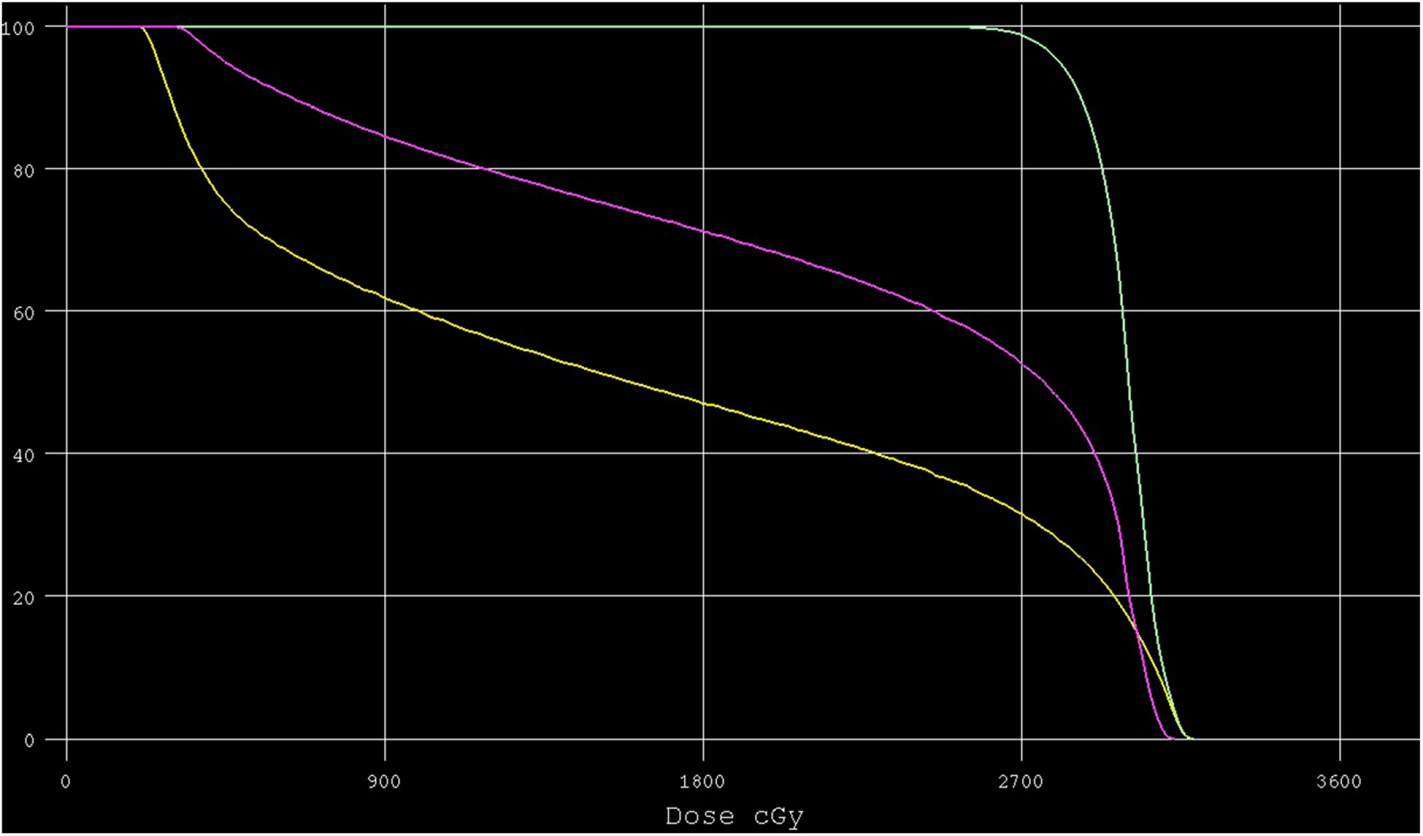
Dose volume histogram of planning target volume, ovary, and uterus. The vertical axis and horizontal axis show the volume and dose, respectively. The ovary and uterus are shown as *yellow* and *pink* lines, respectively

To achieve dose constraints of 12 Gy to her entire ovary, we used horizontally opposed pair fields for radiotherapy to minimize the irradiation to her endometrium and ovary. The irradiated field and dose distribution for this case are shown in Fig. 2a and b, respectively. The clinical target volume (CTV) was defined as the whole rectum and it is shown in orange in Fig. 2a. The planning target volume (PTV) was defined as CTV plus 1 cm margin in all directions and it is shown in green. The ovary and uterus are shown in yellow and pink, respectively. A total dose of 30.6 Gy was given in 17 fractions of 1.8 Gy by 10-Megavolt X-ray linear accelerator to PTV and no chemotherapy was scheduled. As a result, one-third of her uterus and half of her ovary would be outside the irradiation field. A dose volume histogram is show in Fig. 3. The mean dose received by her uterus and ovary was 22.5 Gy and 16.6 Gy, respectively. We determined that a hot area of a maximum of 130% spreading to the soft tissue (subcutaneous fat) was acceptable as the actual dose was 39.8 Gy. Mild radiation dermatitis (Grade 2) around her anus was observed as an acute adverse event.

She completed radiotherapy and was discharged in March 2014, but returned to the Department of Obstetrics and Gynecology in May 2014 due to amenorrhea. A female hormone test revealed a reduction in estrogen level and an increase in follicle-stimulating hormone level. She was thus diagnosed as having premature ovarian failure. Kaufmann therapy was then commenced. After approximately 1 year of treatment, positive pregnancy was confirmed (April 2015), and she revisited the clinic. A gestational sac was confirmed by ultrasound examination, and the second month of pregnancy was diagnosed. Surprisingly, she had conceived naturally. Finally, she safely gave birth to a baby girl without congenital abnormalities in October 2015.

## Discussion

Non-gastric MALT lymphomas can occur in the colon, lung, thyroid gland, salivary glands, mammary gland, and ophthalmologic fields. Primary rectal MALT lymphoma is a particularly rare disease, accounting for less than 1% of all MALT lymphomas [2, 3]. Rectal MALT lymphomas tend to show elevated lesions, such as polyps or submucosal tumors, during endoscopy, and our case presented with similar characteristic polypoid lesions [9]. Because of the low incidence of the disease, conducting large clinical trials is difficult. Therefore, the optimal therapeutic strategy for this type of lymphoma is yet to be established, and therapeutic strategy is currently determined according to the lesion site or clinical stage. In the case of limited stage MALT lymphomas, local therapy, such as radiotherapy or surgery, is generally the principal course of action. However, MALT lymphomas can be well controlled by radiotherapy alone with approximately 30 Gy, and surgery is considered excessive with regard to organ preservation and the postoperative quality of life. The therapeutic strategy used in the present case was radiation therapy, and a total dose of 30.6 Gy was given in 17 fractions to the whole rectum. After 9 months of radiotherapy, our patient was endoscopically examined, and all the lesions had undergone complete remission. No severe gastrointestinal late adverse events, such as rectal bleeding, have yet been observed.

A critical point of this case was our patient’s desire to bear children, and we wanted to meet her desire, as well as gain good control over her rectal MALT lymphoma. However, when radiotherapy is focused onto the pelvic cavity, ovarian dysfunction as an adverse event of radiotherapy becomes a serious problem. Therefore, we performed radiotherapy using horizontally opposed pair fields, which goes against the era of three-dimensional radiotherapy. Our treatment procedure had two purposes. First, we tried to minimize the amount of irradiation exposure to her ovaries. Although she had frozen embryos stored, we wanted to preserve her ovarian function as much as possible in an effort to give her a chance of a natural pregnancy. Second, we attempted to minimize the amount of irradiation exposure to her endometrium, such that we could maximize the chance of normal implantation if she could not become pregnant naturally and needed to use her frozen embryos.

Schuck *et al*. reported that when the whole ovary is irradiated more than 15 Gy, ovarian dysfunction occurs in almost all patients. In addition, when one side of the ovary is preserved, ovarian dysfunction occurs in approximately half of all patients [10]. The mean dose absorbed by the ovary in this patient’s case was 16.6 Gy, although our treatment procedure was successfully achieved by removing half of her ovary from the irradiation field. Therefore, half of her ovary was not completely irradiated, and we expected her ovarian function to be preserved with a probability of 50%. Following our procedure, she developed temporary ovarian dysfunction; Kaufmann therapy, a replacement therapy comprising estrogen and progesterone for ovarian amenorrhea, was commenced. We consider that preserving half of her ovary using horizontally opposed pair fields for radiotherapy was a key benefit in terms of ovulation and natural pregnancy. On the other hand, the tolerance dose of the endometrium is not yet known and, consequently, there is a potential risk of implantation disorders. Because we could not identify her endometrium by CT, the irradiation dose to her endometrium could not be determined using a dose volume histogram. However, we believe that removing one-third of her uterus from the irradiation field was a key contributor to retaining normal implantation, thereby leading to the safe birth of a baby girl. Although the full dose of 30.6 Gy was used to irradiate her vagina, she delivered her baby without any problem. Therefore, we consider a total dose of approximately 30 Gy does not cause adverse effects to the vagina.

## Conclusions

Here we report the case of a 28-year-old woman with a rectal MALT lymphoma who safely gave birth to a baby following 30.6 Gy radiotherapy to her whole rectum. This case involves several rare points. First, there is a low incidence of primary rectal MALT lymphoma among cases of MALT lymphoma. Second, ovarian function could be preserved by removing half of her ovary from the irradiation field using horizontally opposed pair fields. Finally, by removing one-third of her uterus from the irradiation field, we could preserve normal implantation, which enabled her to give birth naturally. We believe that this case is extremely rare and will be of global interest, and we are proud to be able to report the control of rectal MALT lymphoma and the safe birth of a healthy child in this 28-year-old woman.
